# Identification and Expression Analysis of Candidate Odorant-Binding Protein and Chemosensory Protein Genes by Antennal Transcriptome of *Sitobion avenae*

**DOI:** 10.1371/journal.pone.0161839

**Published:** 2016-08-25

**Authors:** Wenxin Xue, Jia Fan, Yong Zhang, Qingxuan Xu, Zongli Han, Jingrui Sun, Julian Chen

**Affiliations:** The State Key Laboratory for Biology of Plant Diseases and Insect Pests, Institute of Plant Protection, Chinese Academy of Agricultural Sciences, Beijing, 100193, China; USDA Agricultural Research Service, UNITED STATES

## Abstract

Odorant-binding proteins (OBPs) and chemosensory proteins (CSPs) of aphids are thought to be responsible for the initial molecular interactions during olfaction that mediate detection of chemical signals. Analysis of the diversity of proteins involved comprises critical basic research work that will facilitate the development of sustainable pest control strategies. To help us better understand differences in the olfactory system between winged and wingless grain aphids, we constructed an antennal transcriptome from winged and wingless *Sitobion avenae* (Fabricius), one of the most serious pests of cereal fields worldwide. Among the 133,331 unigenes in the antennal assembly, 13 OBP and 5 CSP putative transcripts were identified with 6 OBP and 3 CSP sequences representing new *S*. *avenae* annotations. We used qPCR to examine the expression profile of these genes sets across *S*. *avenae* development and in various tissues. We found 7 SaveOBPs and 1 SaveCSP were specifically or significantly elevated in antennae compared with other tissues, and that some transcripts (*SaveOBP8*, *SaveCSP2* and *SaveCSP*5) were abundantly expressed in the legs of winged or wingless aphids. The expression levels of the SaveOBPs and SaveCSPs varied depending on the developmental stage. Possible physiological functions of these genes are discussed. Further molecular and functional studies of these olfactory related genes will explore their potential as novel targets for controlling *S*. *avenae*.

## Introduction

The grain aphid, *Sitobion avenae* (Fabricius) is one of the most important pests of gramineous crops [1]. Damage from their sucking plant sap slows plant growth and reduces the number of tillers, severely diminishing the yield and quality of wheat [2]. In recent years, global climate warming, farming system changes and other factors have contributed to significant enhancement in the reproductive capacity and adaptability of this pest, resulting in great crop damage [3]. Therefore, research on environmentally safe prevention and control strategies for widespread use is extremely important [4, 5]. Detailed analysis of the *S*. *avenae* chemical sensing system can provide insights into aphid olfactory physiology and the molecular mechanisms used to detect semiochemicals. These insights into the chemoreception mechanism of aphids could aid in developing novel olfactory-based control strategies, such as repellents or attractants developing, and provide additional candidate genes for targeted disruption, which could interfere with both plant-aphid interactions and aphid responses of aphid to the external environment.

For aphids, olfaction plays an important role in distinguishing host plant volatiles from other environmental volatiles [6]. Antennae, which are largely the physiological basis for insect chemical ecology, are one of the principle organs that aphids use to recognize chemical information in the environment. Many olfactory-related proteins are responsible for discerning chemical information and regulating and controlling aphid behaviors such as selecting hosts and avoiding natural enemies [7–11]. A variety of odor-related proteins, such as odorant-binding proteins (OBPs), chemosensory proteins (CSPs), odorant receptors (ORs), sensory neuron membrane proteins (SNMPs) and odorant degrading enzymes (ODEs), contribute to the initiation of olfactory perception in insects. OBPs and CSPs, which are water-soluble, globular proteins that are concentrated (as high as 10 mM) in the sensillum lymph of insect antennae [12–17], are thought to provide the initial molecular interactions for chemical signals (semiochemicals) and to ferry the semiochemical molecules through the antennal sensillum lymph to the olfactory receptors (ORs).

Since the first insect OBP was identified from the antennae of male *Antheraea polyphemus* in 1981 [12], many new OBPs have been identified, the discovery of which has been greatly aided by new generation genome and transcriptome analyses. Genome sequencing facilitated the identification of 15 OBP genes in the hemipteran *Acyrthosiphon pisum* [18]. In contrast, mRNA-based transcriptome sequencing has led to the discovery of numerous OBPs from diverse hemipteran pests including *Lygus lineolaris* [19], *Adelphocoris lineolatus* [20], *Aphis gossypii* [21], *Nilaparvata lugens* [22, 23], *Sogatella furcifera* [24], and *Apolygus lucorum* [25]. Interestingly, the number of OBPs reported has differed depending on the species (eg. *A*. *gossypii* has 9 OBPs, *L*. *lineolaris* has 33 OBPs). Based on qPCR analyses, it has become clear that many of the identified OBP genes are highly expressed in antennae [18–25]. However, OBPs are also highly expressed in other tissues, such as legs and heads, which suggests that these OBPs might be associated with taste perception or participate in other physiological functions [26, 27].

OBPs can vary in the amino acid spacing between helices, in the lengths and positions of the loops that connect the helices and in the lengths of the C- and N-termini [28]. The nature and shape of the binding cavities can also differ, presumably representing specificities for different odorant molecules. For assessing the role of OBPs in olfaction *in vitro*, fluorescence competitive binding assays have been used to examine the binding capacity of recombinantly expressed OBPs for specific odorant ligands [15]. Although aphids are a major hemipteran group with numerous species throughout the world, a limited number of ligands have been assayed. The binding properties of three classical pea aphid OBPs (*ApisOBP1*, *ApisOBP3* and *ApisOBP8*) have been investigated using 12 chemical compounds [29]. Research has confirmed that OBPs are keys in the perception of (E)-β-farnesene (EβF), an aphid alarm pheromone widely used in olfaction-based approaches to control aphid populations [30]. The chemical can interfere with an insect’s ability to find suitable hosts or olfactory cues regarding natural enemies, and has been used successfully in the “push–pull” pest control strategy [31, 32]. Different OBPs in various aphid species have been reported to play critical roles in discerning EβF in each respective species. Ligand binding assays suggested that *ApisOBP3* and *SaveOBP7* each have high binding affinities for EβF [29, 33].

Like OBPs, CSPs are also secreted proteins but they have a lower molecular weight (10–16 kDa). The CSP family contributes to the recognition of sex pheromones [34] and general odorants [35] and to other functions such as leg regeneration [36], development [37] and feeding [38]. Transcriptome sequence data for hemipterans are currently limited to 13 CSPs from *A*. *pisum* [18], 11 CSPs from *N*. *lugens* [39] and 9 CSPs from *A*. *gossypii* [21], and their exact functions are largely unknown. Examination of gene expression profiles, particularly in various parts of the body, and phylogenetic analyses could potentially provide important information concerning the function of CSP genes. Two CSPs identified in *Megoura viciae*, MvicOS-D1 and MvicOS-D2 did not bind any of 28 compounds known to elicit an electrophysiological response in electroantennograms or in single olfactory neuron preparations [40].

Our understanding of the molecular components comprising the *S*. *avenae* olfactory system is incomplete both in sequences and expression data. To understand the physiological mechanism underlying volatile detection in wheat aphids better, more research on olfactory related proteins has been needed. In our present work, we used an antennal transcriptome to identify OBPs and CSPs in winged and wingless *S*. *avenae*, and used quantitative real-time PCR to assess their expression in different tissues and ages. In addition, we discuss potential roles of the identified OBPs/CSPs transcripts in olfactory or other physiological processes.

## Materials and Methods

### Ethics statement

Grain aphids (*S*. *avenae*) were from a parthenogenetic colony initially collected from wheat at Langfang Farm, (the experimental farm of the Institute of Plant Protection, Chinese Academy of Agricultural Sciences), Hebei Province, China, which is not privately owned or protected. The grain aphid is neither endangered nor protected so no specific permission was required for its collection.

### Aphid samples

*S*. *avenae* adults were collected from wheat fields at Langfang (39°30′42′′N, 116°36′7′′E), Hebei Province, China, in 2015, and a single wingless aphid was chosen to be reared as a parthenogenetic colony in the laboratory at 22 ± 1°C, with 75% relative humidity and 16h light/8 h dark. After 10 generations, antennae from 1000 winged and 1000 wingless adult aphids of *S*. *avenae* were collected, immediately frozen in liquid nitrogen, and stored at −80°C for transcriptome sequencing. The winged and wingless antennae for transcriptome analysis were collected five times, with ~200 aphids from each sample collected per time. The antennae were pulled off with forceps. Tissues from winged and wingless adults (1000 winged antennae, 1000 wingless antennae, 20 winged heads without antennae, 20 wingless heads without antennae, 20 winged thoraxes, 20 wingless thoraxes, 20 winged abdomens, 20 wingless abdomens, 1000 winged legs and 1000 wingless legs) and whole bodies from each development stage of aphid (15 individuals of 1st instar nymphs or 2nd instar nymphs, 10 of winged or wingless 3rd instar nymphs, 5 of winged or wingless 4th instar nymphs, 5 of winged adults, respectively) were collected in tubes as respective bulked samples with three replicates and immediately frozen in liquid nitrogen and stored at −80°C until needed.

### Transcriptome sequencing, assembly and functional annotation

Antennal total RNA from winged or wingless antennae of *S*. *avenae* was extracted using TRIzol reagent (Invitrogen, Carlsbad, CA, USA) according to the manufacturer’s instructions. After assessing RNA quality on a spectrophotometer NanoDrop 2000c (Thermo Fisher Scientific, Waltham, MA, USA), a 3 μg RNA sample with standard quality ratios (1.8 < OD260/280 < 2.1) was purified using poly-T oligo-attached magnetic beads. Divalent cations under elevated temperature in NEBNext First Strand Synthesis Reaction Buffer (5×) was used for fragmentation. Single-stranded (ss) cDNA was synthesized using a random hexamer primer, M-MuLV Reverse Transcriptase and DNA Polymerase I and RNase H (NEB, USA). The 3' ends of the DNA fragments were adenylated and the NEBNext Adaptor was ligated to the fragments for hybridization. The library fragments were purified with the AMPure XP system (Beckman Coulter, Beverly, MA, USA) to size select cDNA fragments ~ 150–200 bp length. Then 3 μL USER Enzyme (NEB, USA) was used with size-selected, adaptor-ligated cDNA at 37°C for 15 min followed by 5 min at 95°C prior to PCR. PCR was performed with Phusion High-Fidelity DNA polymerase, Universal PCR primers and Index (X) Primer. The products were purified (AMPure XP system), and library quality was assessed using the Agilent Bioanalyzer 2100 system (Agilent Technologies, CA, USA). Clustering of the index-coded samples was performed on a cBot Cluster Generation System using TruSeq PE Cluster Kit v3-cBot-HS (Illumina, China) according to the manufacturer’s instructions. The library preparations were sequenced on an Illumina Hiseq 2500 platform and paired-end reads (the sequencing strategy was PE125) were generated after cluster generation. After sequencing, the raw reads were processed to remove low quality and adaptor sequences by ng_qc, and then assembled into unigenes using Trinity r20140413p1 min_kmer_cov:2 and other default parameters [41]. Then the unigenes were annotated using seven databases, including the non-redundant protein sequence (Nr, e-value = 1e^-5^), non-redundant nucleotide (Nt, e-value = 1e^-5^), Pfam (e-value = 0.01), Clusters of Orthologous Groups (KOG/COG, e-value = 1e^-3^), Swiss-Prot (e-value = 1e^-5^), Kyoto Encyclopedia of Genes and Genomes (KEGG, e-value = 1e^-10^) and Gene Ontology (GO, e-value = 1e^-6^) databases.

### Identification and verification of transcripts encoding putative OBPs and CSPs

We used a motif search program consisting of C1-X_15-39_-C2-X_3_-C3-X_21-44_-C4-X_7-12_-C5-X_8_-C6 for OBPs [42] and C1-X_6-8_-C2-X_16-21_-C3-X_2_-C4 for CSPs [43] and the BLASTx program at the National Center for Biotechnology Information (NCBI, http://blast.ncbi.nlm.nih.gov/Blast.cgi) to confirm putative OBP and CSP genes. The candidate OBPs and CSPs were cloned and sequence validated. TransScript First-Strand cDNA Synthesis SuperMix (Transgen, Beijing, China) was used to synthesize template cDNA. PCR reactions were carried out with 2× *Taq* DNA polymerase (Transgen, Beijing, China) with an initial denaturation at 95°C for 5min; 35 cycles of 94°C for 45 s, 55°C for 30 s, 72°C for 1 min; and a final extension at 72°C for 10 min. The PCR products were gel-purified and subcloned into the pEASY-T1 Sample Cloning Vector (Transgen, Beijing, China) and sequenced using standard M13 primers. Gene-specific primers to clone ORF sequences of each OBP and CSP gene were designed using the program Primer3 (http://primer3.ut.ee/) (S1 Table).

### Comparative analysis of transcripts for putative OBPs and CSPs

For comparing the differential expression of putative genes in the winged and wingless antennal transcriptomes of *S*. *avenae*, the read number for the OBPs and CSPs between different morph antennae of OBPs and CSPs was converted to RPKM (reads per kilobase per million mapped reads), using the formula: RPKM (A) = (1,000,000 × *C* × 1,000) / (*N* × *L*), where RPKM (A) is the expression of gene A, *C* is the number of reads uniquely aligned to gene A, *N* is the total number of reads uniquely aligned to all unigenes, and *L* is the number of bases in gene A. The RPKM method eliminates the influence of gene length and sequencing depth on the calculation of gene expression [44].

### Sequence analysis and phylogenetic tree construction

The putative N-terminal signal peptides and most likely cleavage site were predicted by the SignalP 4.1 Server (http://www.cbs.dtu.dk/services/SignalP/). The amino acid sequence was deduced by the WebLab showorf program (http://weblab.cbi.pku.edu.cn/) and the candidate OBPs and CSPs were aligned using CLUSTAL Omega with default parameters (http://www.ebi.ac.uk/Tools/msa/clustalo/) and then arranged by BOXSHADE 3.21 (http://www.ch.embnet.org/software/BOX_form.html). The candidate OBPs and CSPs from *S*. *avenae* and related sequences from other aphid species were chosen for phylogenetic analysis. After sequences were aligned using ClustalX 2.1 with default gap penalty parameters of gap opening 10 and extension 0.2, all the phylogenetic trees were constructed using the neighbour-joining method implemented in MEGA 5.0 [45] with default settings and 1000 bootstrap replications.

A total of 167 OBP protein sequences from 21 hemipteran species were used for the phylogenetic analysis, sequences used are described in S2 Table and include 13 OBPs from *S*. *avenae* identified in the present study, 1 from *Aphis craccivora*, 2 from *Aphis fabae*, 10 from *Aphis glycines*, 13 from *A*. *pisum*, 9 from *A*. *gossypi*, 1 from *Brevicoryne brassicae*, 1 from *Drepanosiphum platanoidis*, 7 from *Metopolophium dirhodum*, 5 from *Megoura viciae*, 6 from *Myzus persicae*, 5 from *Nasonovia ribisnigri*, 5 from *Pterocomma salicis*, 2 from *Lipaphis erysimi*, 5 from *Rhopalosiphum padi*, 1 from *Tuberolachnus salignus*, 12 from *A*. *lucorum*, 14 from *A*. *lineolatus*, 33 from *L*. *lineolaris*, 10 from *N*. *lugens* and 12 from *S*. *furcifera*. In addition, 51 CSPs from seven hemipteran species were used for the phylogenetic analysis and include 5 CSPs from *S*. *avenae* identified in the present study, 7 from *A*. *gossypi*, 3 from *M*. *persicae*, 8 from *A*. *lucorum*, 8 from *A*. *lineolatus*, 9 from *S*. *furcifera* and 11 from *N*. *lugens*. The SaveOBP and SaveCSP accessions are listed in Table 1. The accession numbers for the other genes are in S2 Table.

**Table 1:** Identified OBPs and CSPs in *S*. *avenae* by antennal transcriptome analysis.

Gene name	ID	Length (AA)	Signal peptide	RPKM		Blastx match						
				W	WL	Name	Species	Accession	Score	QC %	E-value	Identity %
***SaveOBP1***	KU140605	152	1-19aa	1.71	37.43	odorant-binding protein 1	*Acyrthosiphon pisum*	NP_001153526	171	98	7e^-48^	55
***SaveOBP2***	KU140606	243	1-19aa	33.43	49.96	odorant-binding protein 2	*Metopolophium dirhodum*	CAR85639	389	91	9e^-130^	99
***SaveOBP3***	KU140607	141	1-23aa	21.21	24.77	odorant binding protein 3	*Drepanosiphum tanoidis*	AEX65663	284	99	9e^-96^	99
***SaveOBP4***	KU140608	199	1-22aa	1.54	1.4	odorant-binding protein 4	*Acyrthosiphon pisum*	NP_001153530	385	96	2e^-130^	96
***SaveOBP5***	KU140609	221	1-25aa	4.2	47.65	odorant-binding protein 5	*Acyrthosiphon pisum*	NP_001153531	455	99	2e^-157^	98
***SaveOBP6***	KU140610	215	1-19aa	364.1	95.14	odorant binding protein 6	*Aphis glycines*	AHJ80892	370	99	3e^-119^	80
***SaveOBP7***	KU140611	149	1-24aa	2484.7	745.7	odorant-binding protein 7	*Aulacorthum solani*	AHH34994	274	99	3e^-86^	90
***SaveOBP8***	KU140612	162	1-18aa	5.13	10.21	odorant-binding protein 8	*Acyrthosiphon pisum*	NP_001153534	327	99	6e^-112^	98
***SaveOBP9***	KU140613	166	1-24aa	2616.9	1781.3	odorant binding protein 9	*Aphis gossypii*	AGE97639	277	99	2e^-90^	85
***SaveOBP10***	KU140614	143	1-24aa	3299.0	1688.9	odorant-binding protein 10	*Acyrthosiphon pisum*	CAR85637	230	99	4e^-92^	91
***SaveOBP13***	KU140615	112	ND	498.72	449.79	odorant-binding protein 13	*Acyrthosiphon pisum*	CAX63070	226	96	7e^-72^	98
***SaveOBP14***	KU140616	167	1-21aa	1535.81	1145.44	odorant binding protein	*Phenacoccus solenopsis*	ALS31061	85.1	81	3e^-16^	31
***SaveOBP15***	KU140617	175	1-22aa	58.39	37.54	odorant-binding protein 11	*Drosicha corpulenta*	ALV87606	65.5	64	4e^-10^	32
***SaveCSP1***	KU140618	118	ND	1875.7	1217.8	chemosensory protein CSP1	*Sitobion avenae*	AFD20365	233	99	7e^-76^	99
***SaveCSP2***	KU140619	147	1-22aa	57264	47476	chemosensory protein CSP2	*Sitobion avenae*	AFD20367	256	99	7e^-83^	100
***SaveCSP3***	KU140620	138	1-19aa	602.12	494.28	chemosensory protein CSP2	*Aphis gossypii*	ACJ64045	239	99	9e^-76^	91
***SaveCSP4***	KU140621	157	1-25aa	1.83	22.3	chemosensory protein 7	*Aphis gossypii*	AGE97646	281	99	6e^-92^	88
***SaveCSP5***	KU140622	231	1-16aa	143.71	70.44	chemosensory protein-like	*Acyrthosiphon pisum*	NP_001119650	439	99	2e^-148^	92

ND: Not detected; W: winged antennae; WL: wingless antennae; QC: Query cover

### Expression analysis of OBPs and CSPs in different-aged aphids and tissues

Total RNA from different tissues of winged and wingless aphids (antennae, heads without antennae, thoraxes, abdomens and legs) and whole bodies from each stage were extracted using TRIzol reagent (Invitrogen, Carlsbad, CA, USA) according to the manufacturer’s instructions. The ratio of OD260/280 was measured on a spectrophotometer NanoDrop 1000 (Thermo Fisher Scientific, Pittsburgh, PA, USA). Single-stranded cDNA templates using 1 μg RNA from various samples were synthetized using TransScript One-Step gDNA Removal and cDNA Synthesis SuperMix (Transgen, Beijing, China) according to the manufacturer’s instructions.

The expression level of each SaveOBP and SaveCSP transcript across the developmental stages stages and tissues in winged and wingless aphids was assessed using quantitative real-time PCR (qPCR). Specific primer pairs for qPCR were designed with Primer 3 (S3 Table), and qPCR was performed on an ABI 7500 Real-Time PCR System (Applied Biosystems, Carlsbad, CA). Two reference genes, β-actin and NADH dehydrogenase were used for normalizing target gene expression and correcting for sample-to-sample variation [46]. The qPCR reactions were performed in 20 μL reactions containing 10 μL SYBR *Premix Ex Taq* (TaKaRa, Beijing, China), 0.5 μL of each primer (10 μM), 0.4 μL Rox Reference Dye, 2 μL sample cDNA, and 6.6 μL sterilized H_2_O. The qPCR cycling parameters were 95°C for 30 s, followed by 40 cycles of 95°C for 15 s and 60°C for 30 s. Next the PCR products were heated to 95°C for 15 s, cooled to 60°C for 1 min and 95°C for 15 s to measure the dissociation curves. Negative controls without a template were included in each experiment to check reproducibility; each qPCR reaction for each sample was done in three technical replicates and three biological replicates for each transcript. Standard curves for reference genes and candidate genes were generated by gradient dilution to identify proper primers with 90–110% amplification efficiency and without nonspecific amplification. Relative quantities were calculated using the Vandesompele Method [47]. Differences in transcript expression in various tissues and ages were statistically analyzed with a one-way ANOVA using SAS 9.1 (SAS Institute, Cary, NC, USA) followed by the least-significant difference (LSD) method.

## Results

### Overview of transcriptomes

A total of 2.22 and 2.30 million raw reads were obtained from *S*. *avenae* antennae libraries from winged and wingless aphids, respectively. After removal of low-quality, adaptor, and contaminating sequences, 2.14 and 2.23 million clean reads were retained and assembled into 147,665 distinct transcripts (mean length = 652 bp) and 133,331 unigenes (mean length = 594 bp). The length distribution can be seen in S1 Fig.

In total, 89,452 (67.06% of all 133,331 unigenes), 42,393 (31.79%), 54,572(40.92%), 39,254 (29.44%), 55,639 (41.72%), 32,300 (24.22%) and 60,371 (45.27%) transcripts from *S*. *avenae* were annotated using the Nr, Nt, Pfam, KOG/COG, Swiss-Prot, KEGG and GO databases respectively (S4 Table).

In the GO annotation, biological process, cellular process and metabolic process were the most abundant GO terms. The cluster for biological process was the next largest group. Most transcripts that corresponded to molecular function were related to binding and catalytic activity (S2 Fig). In the KOG classification, unigenes clustered into 26 categories (S3 Fig). Among these categories, general function prediction was the dominant category, followed by signal transduction and post-translational modification, protein turnover and chaperon. All the unigenes annotated in the KO database were assigned to the 5 biological pathways described in the KEGG database: cellular processes, environmental information processing, genetic information processing, metabolism, and organismal systems (S4 Fig). The most common pathway was metabolism followed by genetic information processing, organismal systems and cellular processes. In the environmental information processing group, most genes (2776) were involved in signal transduction.

### Identification and analysis of OBP genes in *S*. *avenae*

**Fig 1:**
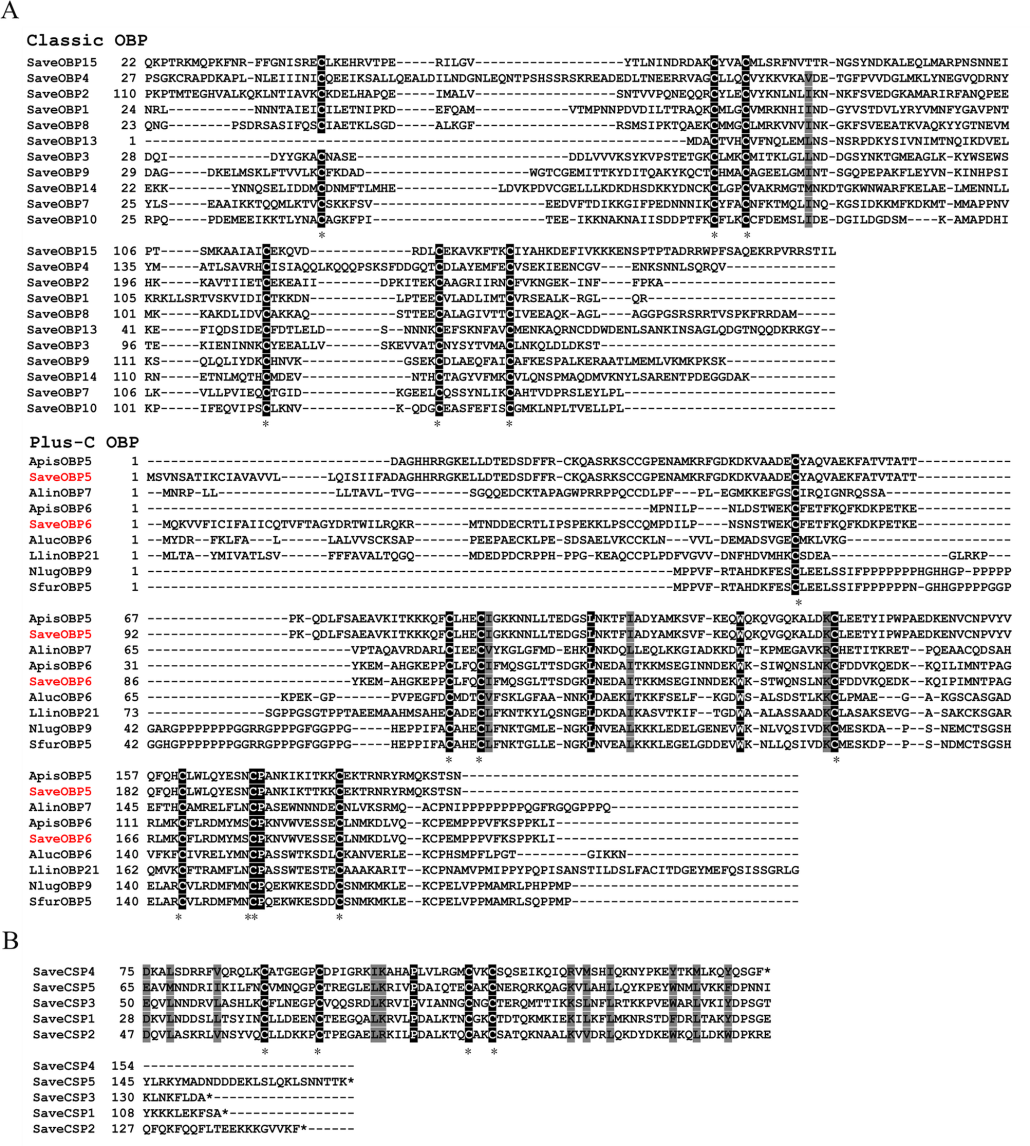
Alignment of amino acid sequences of the OBPs and CSPs in *S*. *avenae*. Sequences were aligned by Clustal Omega and edited using BoxShade. Black boxes show conserved cysteines. The conserved Cys residues are indicated. Shading represents sequence identity > 70%.

Thirteen putative OBPs were identified (Table 1) using a motif search and the NCBI BLASTx program. We named the OBP genes *SaveOBP1* to *10* and *SaveOBP13* to *15*, following the nomenclature established for *A*. *pisum* [18, 21]. All OBP transcripts were confirmed by molecular cloning and sequencing. The sequencing results showed no differences with the trancriptomic data. Apart from *SaveOBP13*, the other 12 OBPs had complete open reading frames (ORFs) consisting of 400–750 bp nucleotides. Among the 13 OBPs, all have the characteristic insect OBP sequence motif [25] (Fig 1A). 9 SaveOBPs (SaveOBP2–3, 7–10 and 13–15) had the classic hemipteran OBP Cys motif (C1-X_22-32_-C2-X_3_-C3-X_36-46_-C4-X_8-14_-C5-X_8_-C6) [48]. *SaveOBP4* had 49 amino acids between the first and second conserved cysteines and 21 amino acids between the fourth and fifth conserved cysteines. *SaveOBP1* had 47 amino acids between the third and fourth conserved cysteines. 2 SaveOBPs (*SaveOBP5* and *SaveOBP6*) belong to the ‘Plus-C’ OBP family and have the similar motif to the Cys spacing pattern C1-X_20-41_-C2-X_3_-C3-X_41-46_-C4-X_19-29_-C4a-X_9_-C5-X_8_-C6-P-X_9-10_-C6a-X_9-10_ [49] (Fig 1A). The signal peptide predictions are shown in Table 1. The 13 OBPs shared 6.21–30.24% amino acid identities with each other (S5 Table).

**Fig 2:**
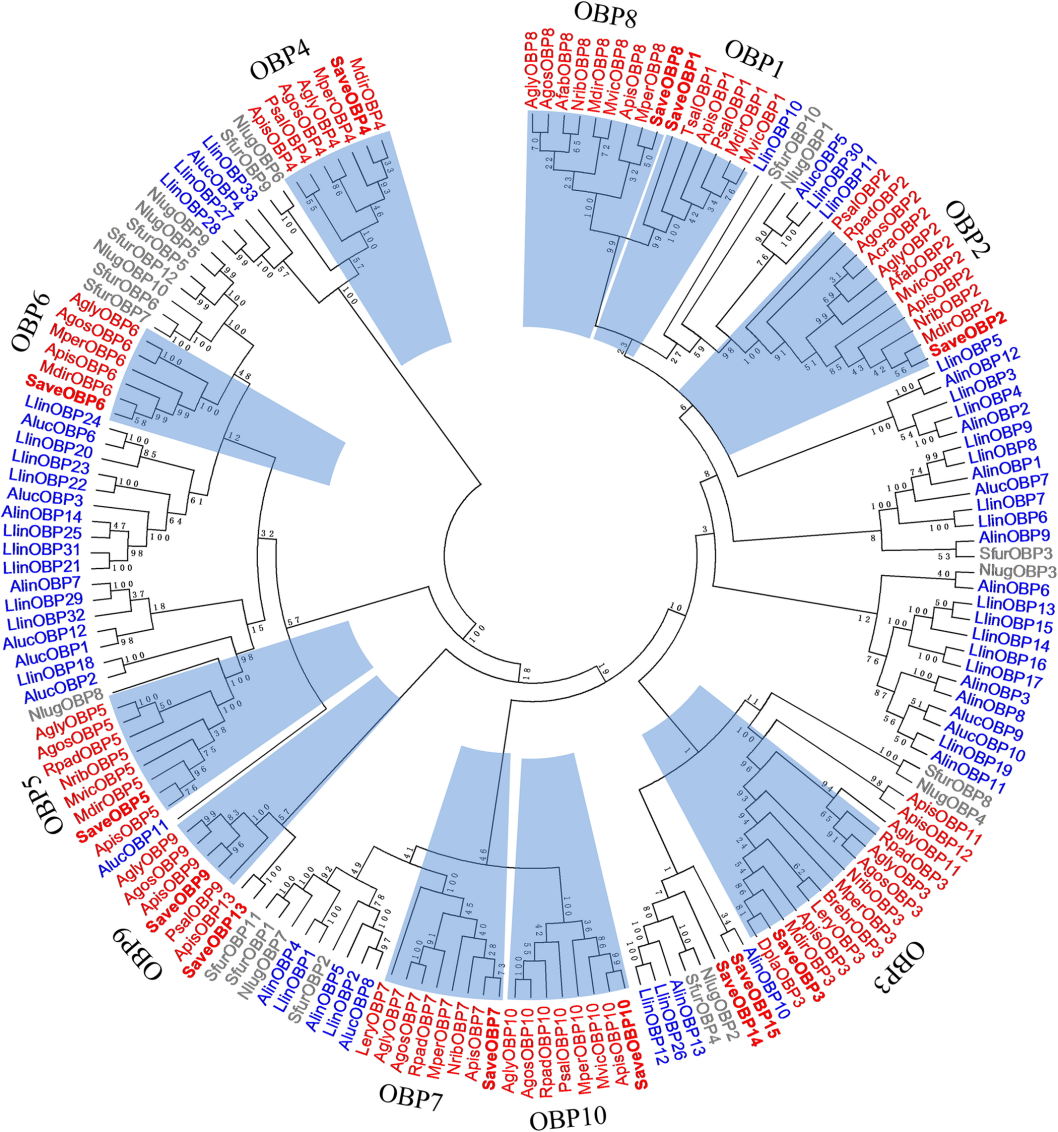
Phylogenetic tree of 167 OBPs from 21 hemipteran species. The tree was constructed using MEGA 5.0 with bootstrap support based on 1000 iterations. Aphid sequences are in red. Bug sequences are in blue. Planthopper sequences are in gray. Major clades for aphid OBPs are marked in a different color. Abbreviation for these aphid species: Save, *S*. *avenae*; Acra, *A*. *craccivora*; Psal, *P*. *salicis*; Brebr, *B*. *brassicae*; Apis, *A*. *pisum*; Dpla, *D*. *platanoidis*; Mper, *M*. *persicae*; Nrib, *N*. *ribisnigri*; Rpad, *R*. *padi*; Mvic, *M*. *viciae*; Tsal, *T*. *salignus*; Afab, *A*. *fabae*; Agos, *A*. *gossypii*; Mdir, *M*. *dirhodum*; Lery, *L*. *erysimi*; Agly, *A*. *glycines;* Aluc, *A*. *lucorum*; Alin, *A*. *lineolatus*; Llin, *L*. *lineolaris*; Nlug, *N*. *lugens*; Sfur, *S*. *furcifera*.

The phylogenetic tree for the 165 OBPs from bugs, aphids and planthoppers, revealed diverging relationships. Aphid OBPs clustered into 10 major groups, each containing several homologous OBPs from different aphid species [26]; amino acid identities for each group (OBP1–10) were 79.17%, 90.09%, 86.78%, 88.45%, 84.10%, 78.68%, 73.84%, 89.30%, 86.75% and 91.94%, respectively, indicating high conservation among the different aphid species. Most of the orthologous sequences in the *S*. *avenae* OBPs were largely limited to aphid OBPs with an average bootstrap value of 94%, and 4 had a high degree of similarity with *A*. *pisum* (Fig 2), which suggested that these sequences might have vertically descended from the same ancestors and were conserved for common functions in aphids. *SaveOBP14* and *SaveOBP15* were clustered with OBPs of bugs and planthoppers.

### Identification of CSP genes in *S*. *avenae*

In total, we identified five transcripts belonging to the CSP family (Table 1); *SaveCSP1*–*5*. By BLASTx comparative analysis, all these genes were most similar to CSP sequences from aphids. All of the CSP transcripts were confirmed by molecular cloning and sequencing, showing that there was no difference between the transcriptome sequencing result and the sequence clones. The CSPs had structural features typical of insect CSPs with four conserved cysteines fitting the hemipteran Cys spacing motif C1-X_5-6_-C2-X_18-19_-C3-X_2_-C4 (Fig 1B) [48]. Only *SaveCSP1* did not have a complete ORF, whereas the other four CSPs varied in length from 400–700 bp. The five CSPs shared 11.89–30.82% amino acid identities (S6 Table). The predicted results of the signal peptides are shown in Table 1.

**Fig 3:**
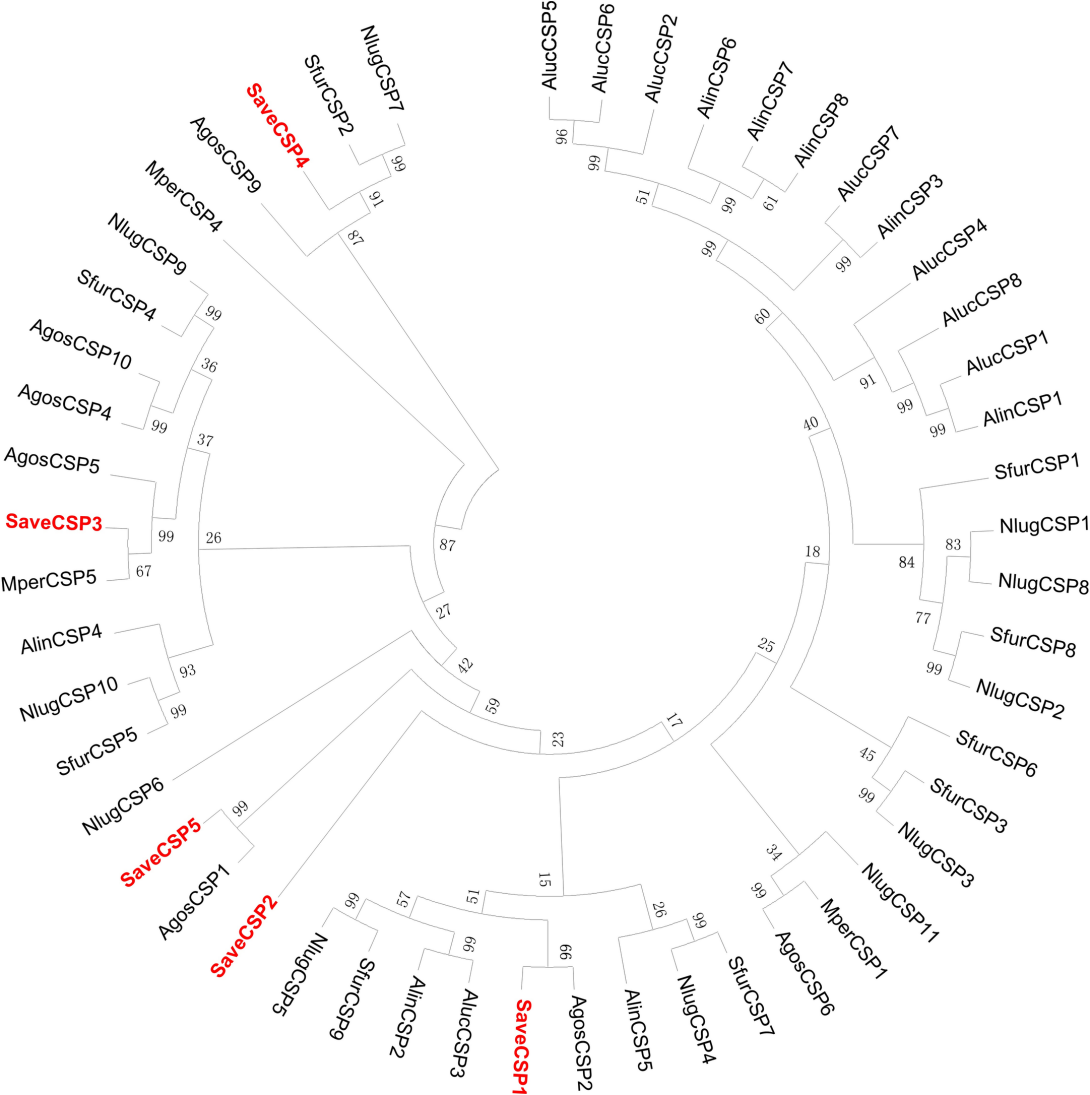
Phylogenetic tree of 51 CSPs from 7 hemipteran species. The tree was constructed using MEGA 5.0 with bootstrap support based on 1000 iterations. SaveOBP sequences are in bold. Abbreviation of these aphid spaces are as follows: Save, *S*. *avenae*; Mper, *M*. *persicae*; Agos, *A*. *gossypii*; Aluc, *A*. *lucorum*; Alin, *A*. *lineolatus*; Nlug, *N*. *lugens*; Sfur, *S*. *furcifera*.

To assign functions to each of the SaveCSPs, we constructed a phylogenetic tree using 51 identified CSPs from seven hemipteran species. *SaveCSP1*, *SaveCSP3* and *SaveCSP5* clustered with aphid CSPs with an amino acid identity of 65.67%, 95.68% and 59.51%, respectively (Fig 3). At present, there are few studies on aphid CSP genes in hemipteran species.

### Expression profile analysis of SaveOBPs

**Fig 4:**
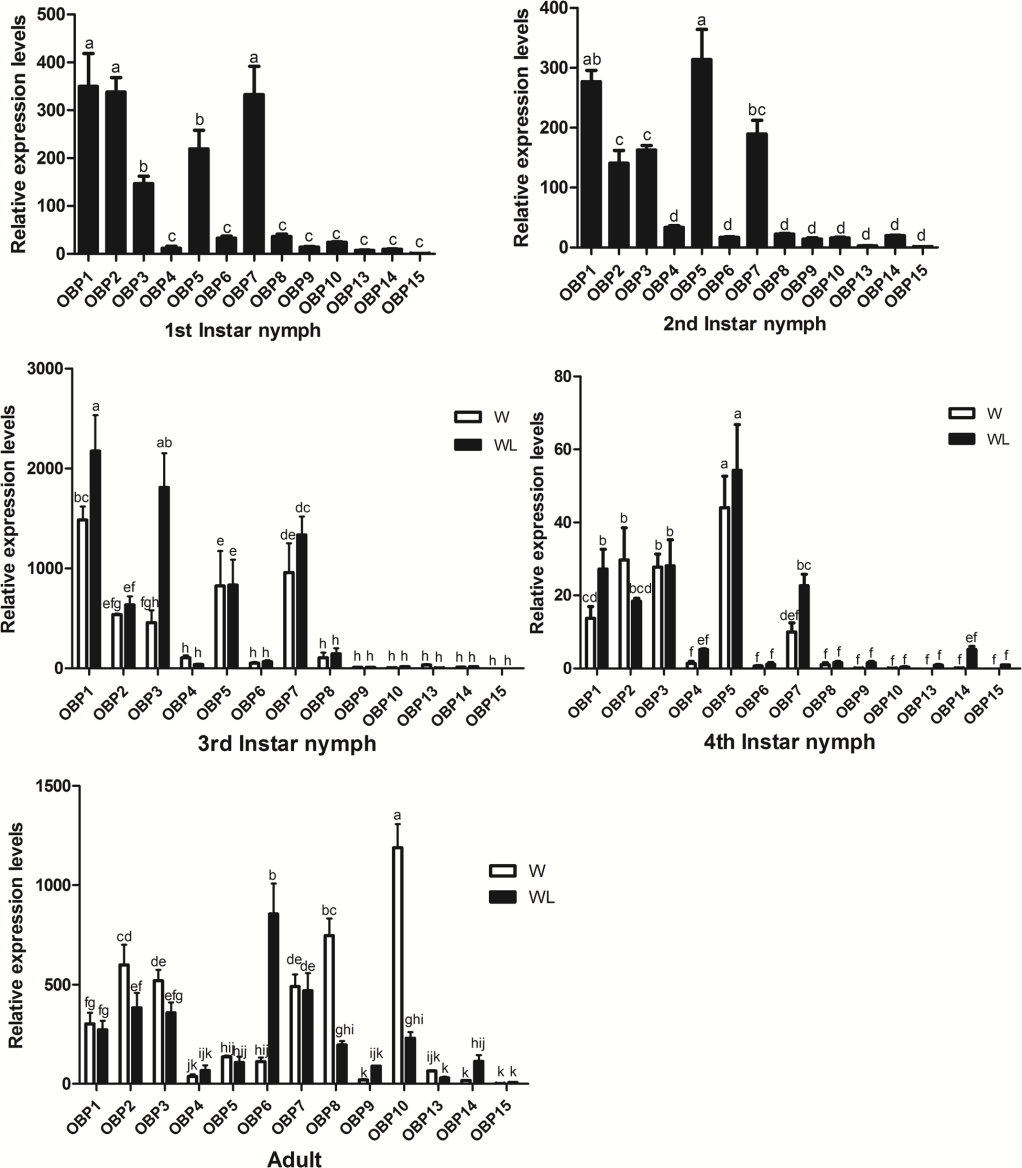
Expression profiles of candidate OBPs in each age of *S*. *avenae*. Fold changes for 1st or 2nd instar nymphs are relative to transcript levels of *SaveOBP15*. Fold-changes for other stages are relative to transcript levels of wingless *SaveOBP15* in wingless aphids of the same age. Differences in mean transcript levels were compared using one-way ANOVA, followed by the least-significant difference (LSD) method. Bars with different letters indicate significant differences (*p* < 0.05).

**Fig 5:**
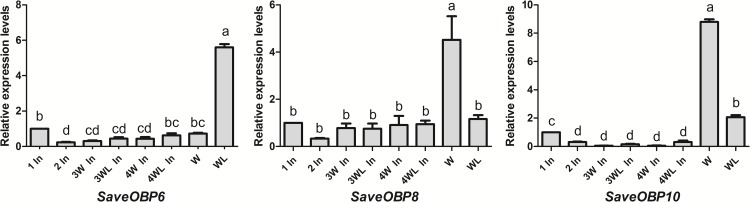
Expression profiles of *SaveOBP6 SaveOBP8* and *SaveOBP10* in different ages of *S*. *avenae*. Fold-changes are relative to transcript levels in 1st instar nymphs. Differences in mean transcript levels were compared using one-way ANOVA, followed by the least-significant difference (LSD) method. Bars with different letters indicate significant differences (*p* < 0.05). 1 In: 1st instar nymph; 2 In: 2nd instar nymph; 3W In: 3rd winged instar nymph; 3WL In: 3rd wingless instar nymph; 4W In: 4th winged instar nymph; 4WL In: 4th wingless instar nymph; W: winged adult; WL: wingless adult.

The results of qPCR showed that the expression levels of *SaveOBP1*, *SaveOBP2*, *SaveOBP3*, *SaveOBP5* and *SaveOBP7* genes were higher than those of other OBPs in all instars (Fig 4). Expression of *SaveOBP8* and *SaveOBP10* both increased when aphids became winged adults. At the same time, expression of *SaveOBP6* increased when aphids became wingless adults (Fig 5).

**Fig 6:**
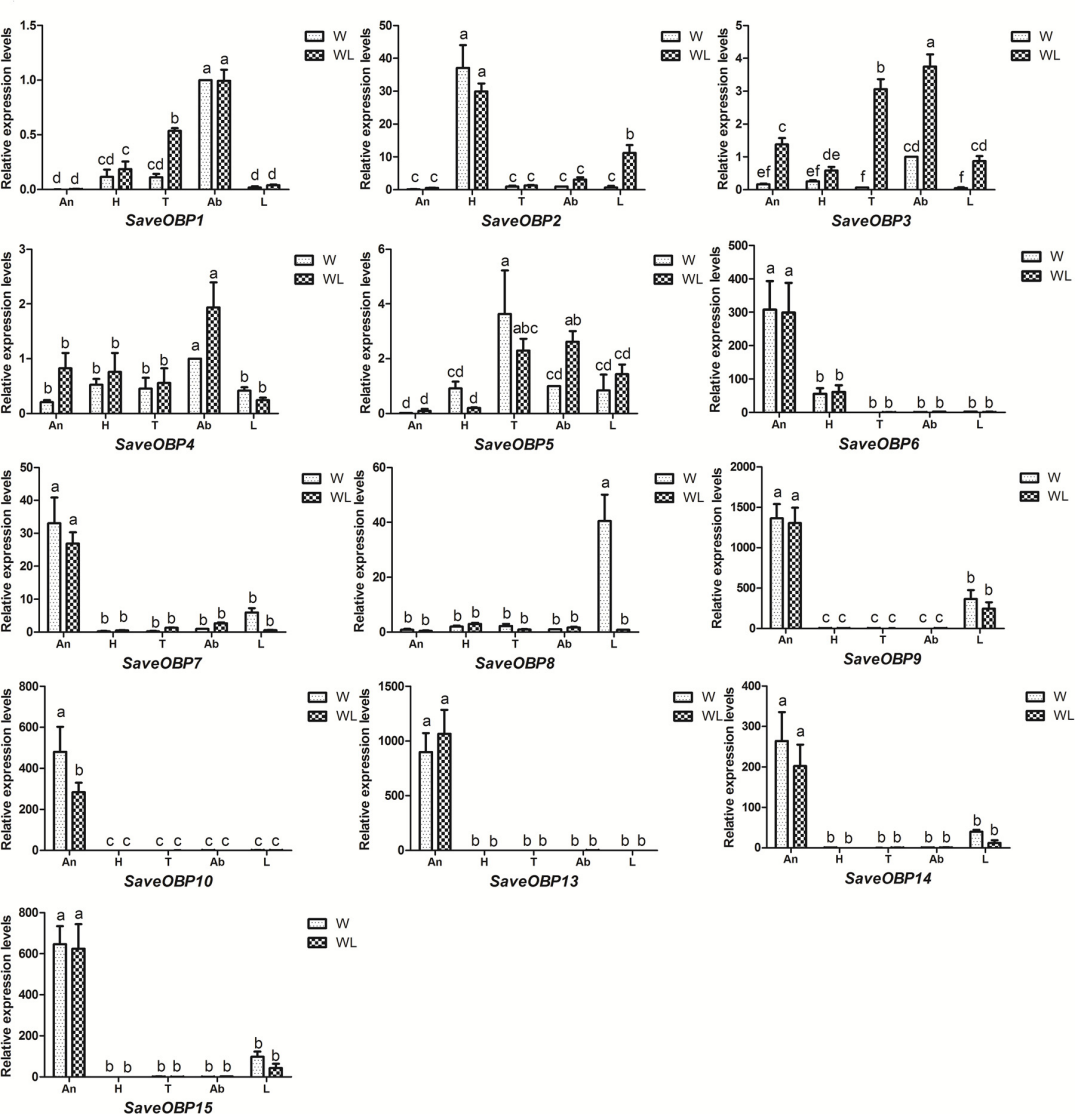
Tissue expression profiles of candidate OBPs in *S*. *avenae*. Fold-changes are relative to transcript levels in abdomens of winged adult aphids. Differences in mean transcript levels were compared using one-way ANOVA, followed by the least-significant difference (LSD) method. Bars with different letters indicate significant differences (*p* < 0.05). An: antennae, H: heads, T: thoraxes, Ab: abdomens, L: legs.

Seven SaveOBP transcripts (*SaveOBP6*, *SaveOBP7*, *SaveOBP9*, *SaveOBP10*, *SaveOBP13*, *SaveOBP14* and *SaveOBP15*) were specifically or highly expressed in antennae of both winged and wingless aphids. Based on RPKM, these seven genes were more abundant than the other SaveOBPs in winged and wingless antennae. Among 13 SaveOBP genes, *SaveOBP10* had the highest RPKM value, followed by *SaveOBP9*, *SaveOBP7*, *SaveOBP14*, *SaveOBP13*, *SaveOBP6* and *SaveOBP15* (Table 1). Expression of *SaveOBP10* in the antennae of winged aphids was higher than in those of wingless aphids (Fig 6).

We also found that expression of *SaveOBP1* was significantly higher in the abdomens of both winged and wingless aphids than in other tissues. *SaveOBP2* was primarily expressed in heads. *SaveOBP3* was significantly elevated in wingless thorax/abdomen. The expression of *SaveOBP8* in the legs of winged aphids was remarkably high (>40 fold higher than in other tissues) (Fig 6).

### Expression profile analysis of SaveCSPs

**Fig 7:**
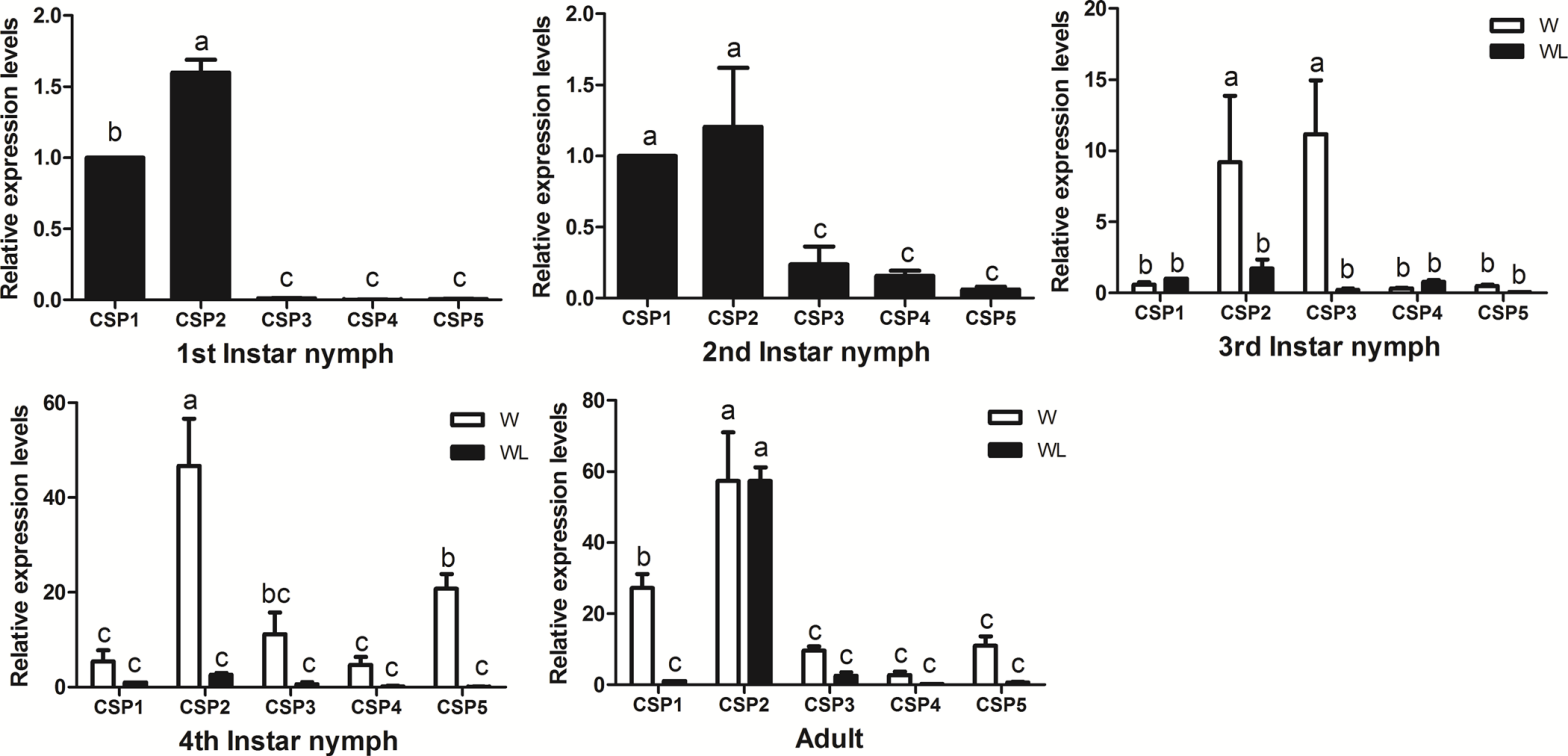
Expression profiles of candidate CSPs in each age of *S*. *avenae*. Fold-changes for 1st and 2nd instar nymphs are relative to the transcript levels of *SaveCSP1*. Fold-changes for other stages are relative to transcript levels of SaveCSP1 in wingless aphids of the same stage. Differences in mean transcript levels were compared using one-way ANOVA, followed by the least-significant difference (LSD) method. Bars with different letters indicate significant differences (*p* < 0.05).

**Fig 8:**
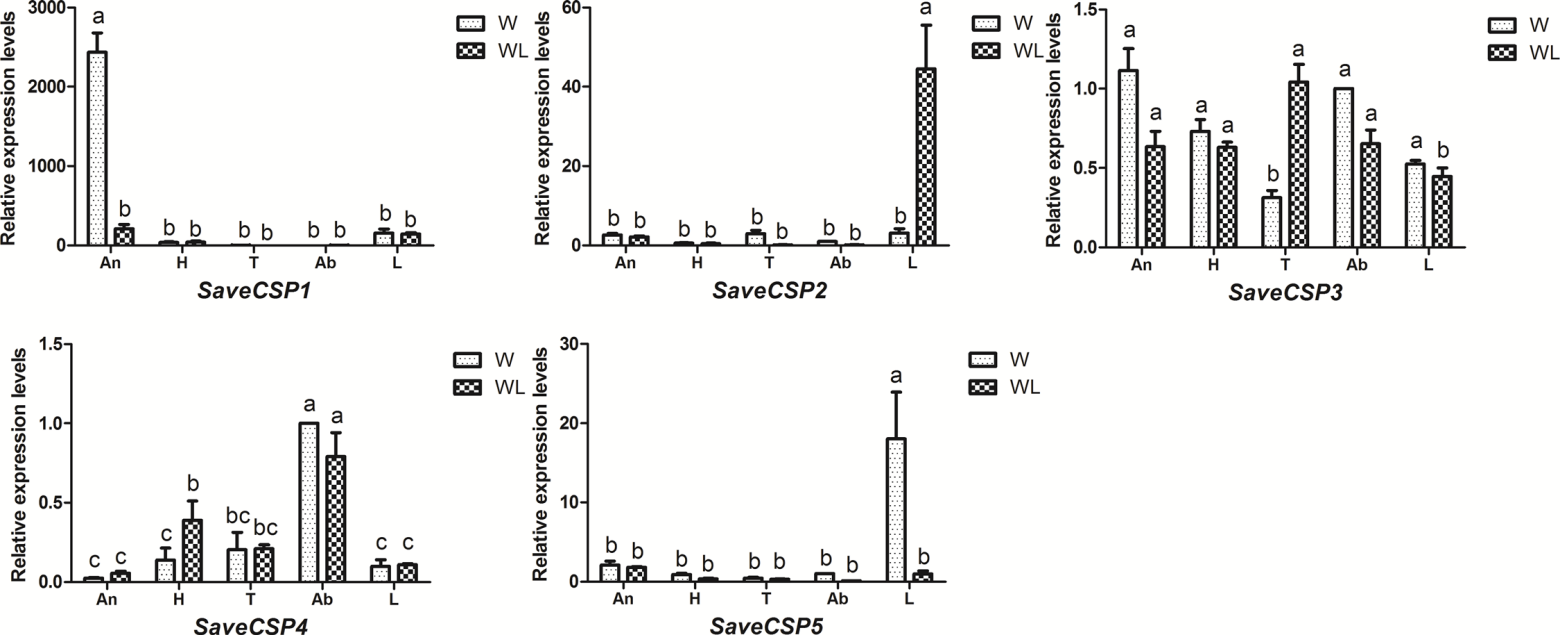
Tissue expression profiles of candidate CSPs in *S*. *avenae*. Fold-changes are relative to transcript levels in abdomens of winged adult aphids. Differences in mean transcript levels were compared using one-way ANOVA, followed by the least-significant difference (LSD) method. Bars with different letters indicate significant differences (*p* < 0.05). An: antennae, H: heads, T: thoraxes, Ab: abdomens, L: legs.

In the developmental qPCR analysis of the five SaveCSPs across each stage (Fig 7), *SaveCSP1* and *SaveCSP2* had the highest expression level in 1st and 2nd instar nymphs. Expressions of *SaveCSP2* and *SaveCSP3* were higher in winged 3rd instar nymphs than in wingless 3rd instar nymphs. For 4th instar nymphs, the expression of *SaveCSP2* and *SaveCSP5* in winged nymphs was higher than in wingless nymphs. *SaveCSP2* was significantly higher in winged and wingless adults. The only difference between winged and wingless adults was *SaveCSP1*, which had higher expression in the antennae of winged adult aphids (>200 fold higher than in the antenna of wingless aphids). *SaveCSP2* was highest in legs of wingless adults, whereas *SaveCSP*5 was highest in the legs of winged aphids. *SaveCSP4* was significantly higher in abdomens of winged and wingless adults (Fig 8).

## Discussion

In this study, we report on the sequencing, assembly and partial annotation of anntenal transcriptomes for winged and wingless grain aphids (*S*. *avenae*) and we identified 13 OBPs and five CSPs. Among them, six OBPs and three CSPs are newly reported transcripts. The number of OBPs is comparable to the 15 for *A*. *pisum* [18], 9 for *A*. *gossypii* [21], 10 for *N*. *lugens* [22] and 12 for *S*. *furcifera* [24]. However, nearly twice as many OBPs have been reported for *A*. *lucorum* (38 OBPs) and *L*. *lineolaris* (33 OBPs) [19, 25]. For CSPs, *S*. *avenae* is fewer than the other species in hemipteran, for example, 13 in *A*. *pisum* [18] and 9 in *A*. *gossypii* [21]. Surprisingly, both OBP11 and OBP12 which were found in *A*. *pisum* were not found in our *S*. *avenae* antennal transcriptome. This similar phenomenon also appeared in the antennal transcriptome analysis of *A*. *gossypii* in which OBP1 and CSP3 were not found [21]. The comprehensive factors of a complicated and heterogeneous host environment are likely responsible for these differences [18] or obvious seasonal alterations in host ranges might lead to the different number of genes for additional and more complex functions [50]. In addition, the cDNA libraries were constructed from antennae total RNA, genes with other functions expressed in other tissues might not appear, and some OBPs and CSPs may not yet have been identified in *S*. *avenae* as a result of the limited ESTs that were sequenced [21].

In the phylogenetic tree, most identified OBPs were clustered in highly conserved groups from different aphid species; the same family of OBPs segregated into different central clusters, distributed equally throughout the phylogenetic tree [51]. The conservation of the CSPs among different aphids might be low, only one of five SaveCSPs, *SaveCSP3*, clustered with *MperCSP5* with over 70% identity in an amino acid sequence. The distribution of candidate orthologs in other aphid species suggests that these genes originating from a common ancestor may have similar functions or that they acquired novel functions via subfunctionalization [25]. These results suggest that OBP and CSP proteins in hemipteran insects undergo extensive gene duplication and divergence by natural selection, strongly indicating that they have diverse functions [24].

Antennae-restricted expression combined with age-restricted expression is a useful criterion to identify genes involved in specific chemoreception functions. Our study revealed that consistently high expression of *SaveOBP1*, *SaveOBP2*, *SaveOBP3*, *SaveOBP5* and *SaveOBP7* in all aphid instars suggests that the respective proteins have a basic conserved role, such as in feeding or identifying general volatiles. High expression of *SaveOBP6* in wingless adults might suggest a functional role in the discrimination of egg-laying substrates and the perception of molecules related to new host-plant location [22]. *SaveOBP8* and *SaveOBP10* with high expression in winged adults might be involved in the perception of molecules related to new host-plant location [52].

Hemipterans typically have a high percentage of antennal expressed OBPs, for example, 5 of 9 OBPs in *A*. *gossypii* [21], 12 of 14 OBPs in *A*. *lineolatus* [20], at least 6 of 10 OBPs in *N*. *lugens* [22], and 21 of 33 OBPs in *L*. *lineolaris* [19]. Seven of 13 SaveOBPs (*SaveOBP6*, *SaveOBP7*, *SaveOBP9*, *SaveOBP10*, *SaveOBP13*, *SaveOBP14* and *SaveOBP15*) were uniquely or primarily expressed in antennae compared with other tissues, indicating a vital olfactory role for these genes.

*SaveOBP2* was dominantly expressed in heads of both winged and wingless aphids; it could be involved in gustatory function in insects [26, 27]. The elevated expression of SaveOBP1 in the abdomens of winged and wingless aphids could indicate a role in the storage and release of chemical compounds from specialized glands localized in the abdomen [25]. OBPs expressed in taste sensilla on legs have been reported to have a role in the behavioral adaptation of *Drosophila sechellia* [53, 54]. *SaveOBP8* was abundantly expressed in legs of winged aphids, which could be related to the adaptation of *S*. *avenae* during migration.

According to previous reports on aphid olfaction, some SaveOBP genes showed the had expression profiles that were similar to orthologous genes in *A*. *gossypii* and *A*. *pisum*. *SaveOBP6*, *AgosOBP6* and *ApisOBP6* clustered in a branch with sequences highly expressed in antennae, which strongly suggests that these genes have the same or similar function [21, 55]. The same phenomena can also be found in the branches of *SaveOBP7* and *ApisOBP7*, *SaveOBP9* and *AgosOBP9*, *SaveOBP10* and *AgosOBP10* [21].

Synthesis of such a high concentration (up to 10 mM) of OBP proteins in insect antennae will consume a lot of energy, so the OBPs should also have some important physiological functions in addition to participating in initial recognition of olfactory signals. On the other hand, OBP expression in non-olfactory tissues has already been ascertained and suggests that they also may function as carriers of chemicals during developmental and physiological processes [25, 56–61]. Overall, OBPs have a very complex expression profile, both relative to body tissues and developmental stages, presumably in connection with their different roles in aphid behaviors [25].

These broad and diverse expression patterns also suggest that different CSPs serve varied functions, including chemosensation [62] and development [63], as well as other processes [37]. *SaveCSP1*, which was highly expressed in the antennae, could be involved in insect chemoreception. *SaveCSP2* and *SaveCSP5*, both of which are higher expressed in legs, might participate in the process of taste or volatile reception or be indicative of olfactory sensilla on the legs [53, 54]. *SaveCSP4* was widely expressed in chemosensory and non-chemosensory tissues. According to the phylogenetic tree, *SaveCSP4* grouped in a clade with *NlugCSP7*, which suggests these two genes may have similar functions in physiological processes other than olfaction [39]. The expression levels of CSPs were affected by age, although without a clear pattern.

Considering all the quantitative qPCR results, it is clear that the olfactory system in winged adults of *S*. *avenae* differs across development stages. For example, *SaveOBP10* and *SaveCSP1* have relatively higher expression levels in winged adult antennae than in other tissues. In addition, expression of *SaveOBP10* was highest in winged adults compared to the other developmental stages. *SaveOBP8* and *SaveCSP5* were also expressed abundantly in legs of winged aphids, about 40- and 20- fold more than in other tissues of winged or wingless aphids.

In summary, using next generation sequencing data for *S*. *avenae*, we identified 13 SaveOBP and 5 SaveCSP transcripts. The comprehensive comparison of expression patterns forms a basis for functional studies, especially in revealing major olfactory organ expression of OBP and CSP genes. On the basis of these data, biochemical analyses and behavioral studies will be done to better understand the significant diversity in the functional roles of one or more olfactory genes in the perception of a specific odor. A better understanding of the insect olfactory system and possible targets for insect pest control [47] should inform searches for eco-friendly pest control alternatives to conventional pesticides.

## Supporting Information

S1 Fig Length distribution of transcripts and unigenes. (TIF)

S2 Fig Gene function classification (GO) of unigenes. (TIF)

S3 Fig KOG classification of unigenes. (TIF)

S4 Fig KEGG pathway classification of unigenes. A: Cellular processes; B: Environmental information processing; C: Genetic information processing; D: Metabolism; E: Organismal systems.
(TIF)

S5 Fig OBP and CSP bands after qPCR in *S*. *avenae*. (TIF)

S1 Table Primers for cloning. (DOCX)

S2 Table GenBank accession numbers of amino acid sequences used to construct the phylogenetic tree. (DOCX)

S3 Table Primers for qPCR. (DOCX)

S4 Table Success rate of gene annotation. (DOCX)

S5 Table Sequence identity between SaveOBPs. Calculations are based on amino acid sequence alignment by DNAMAN. The percentage identity for each pair is shown.
(DOCX)

S6 Table Sequence identity between SaveCSPs. Calculations are based on amino acid sequence alignment by DNAMAN. The percentage identity of each pair is shown.
(DOCX)
