# Supplementary figures and images for: Identification and Expression Analysis of Candidate Odorant-Binding Protein and Chemosensory Protein Genes by Antennal Transcriptome of Sitobion avenae
Source: PLoS One. 2016 Aug 25;11(8):e0161839. doi: 10.1371/journal.pone.0161839 (PMC4999175; doi:10.1371/journal.pone.0161839)

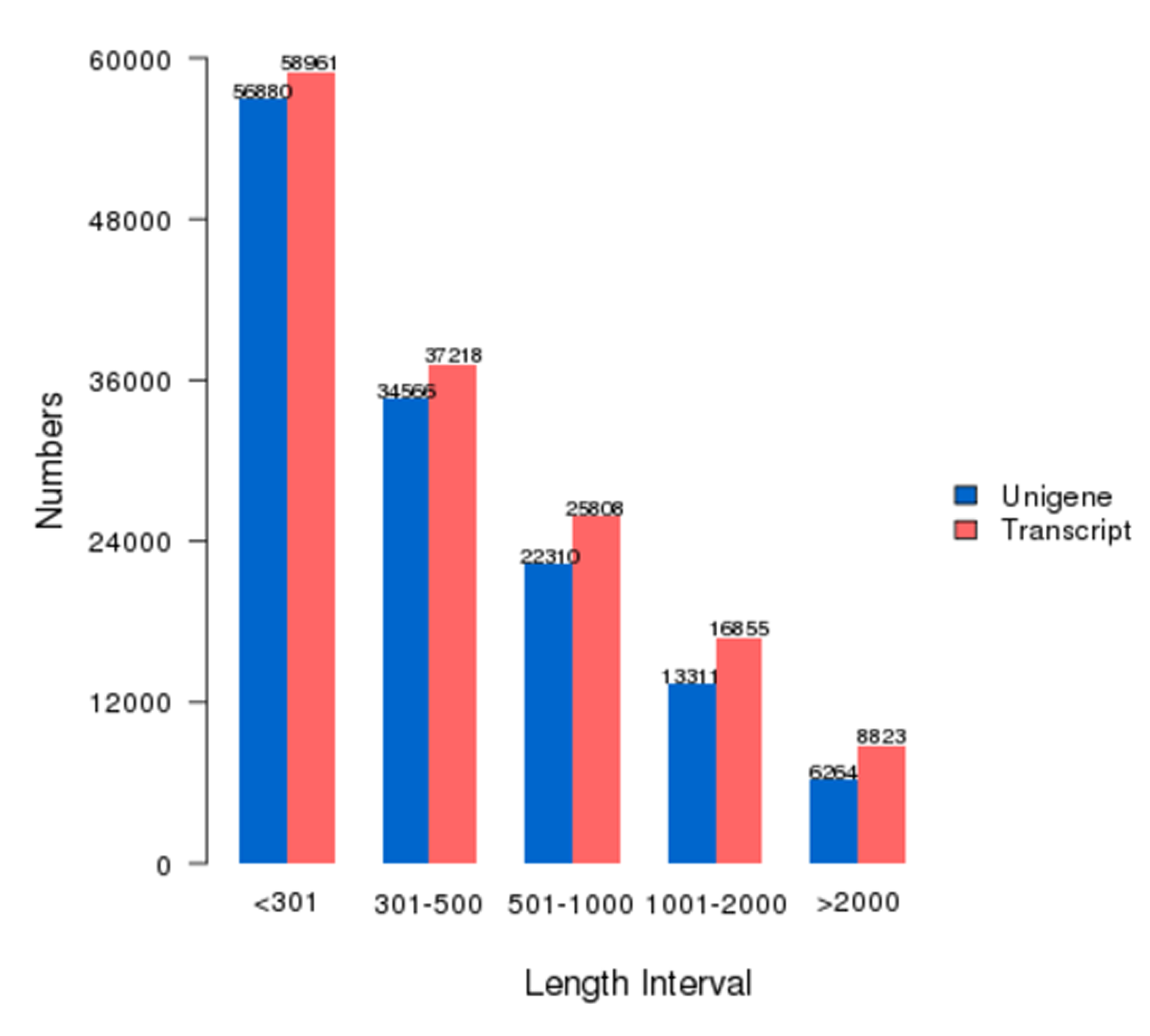

Supplement: S1 Fig — (TIF) [file pone.0161839.s001.tif]

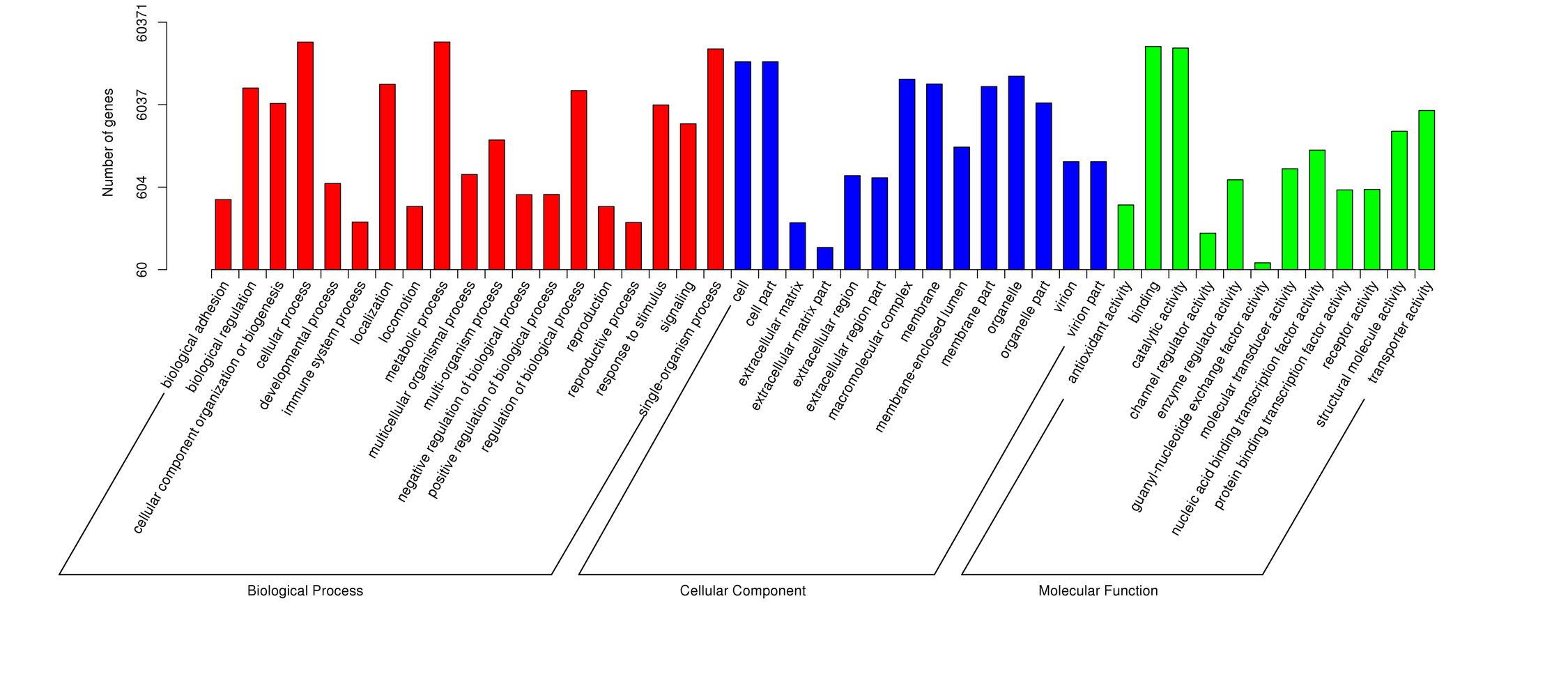

Supplement: S2 Fig — (TIF) [file pone.0161839.s002.tif]

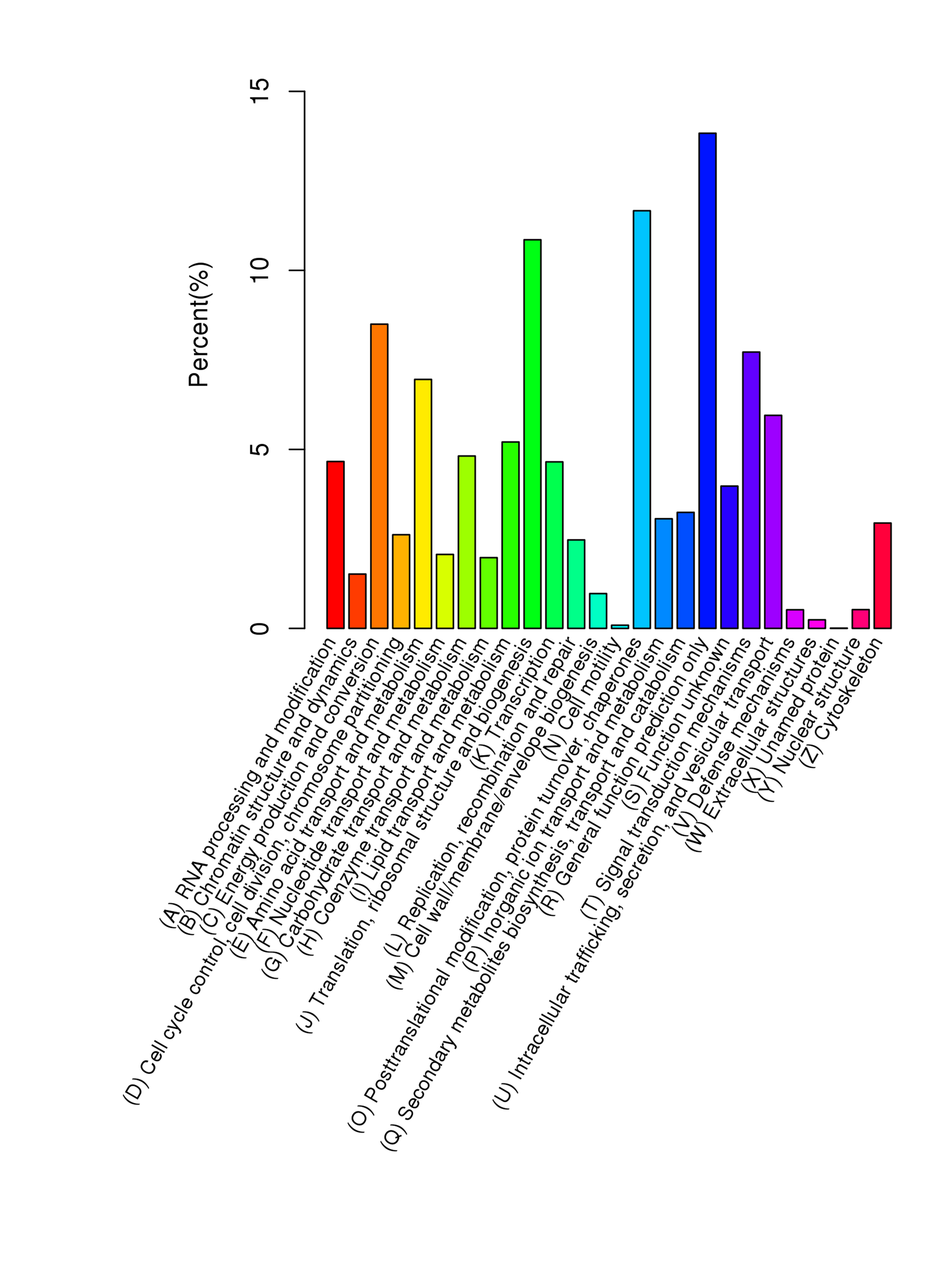

Supplement: S3 Fig — (TIF) [file pone.0161839.s003.tif]

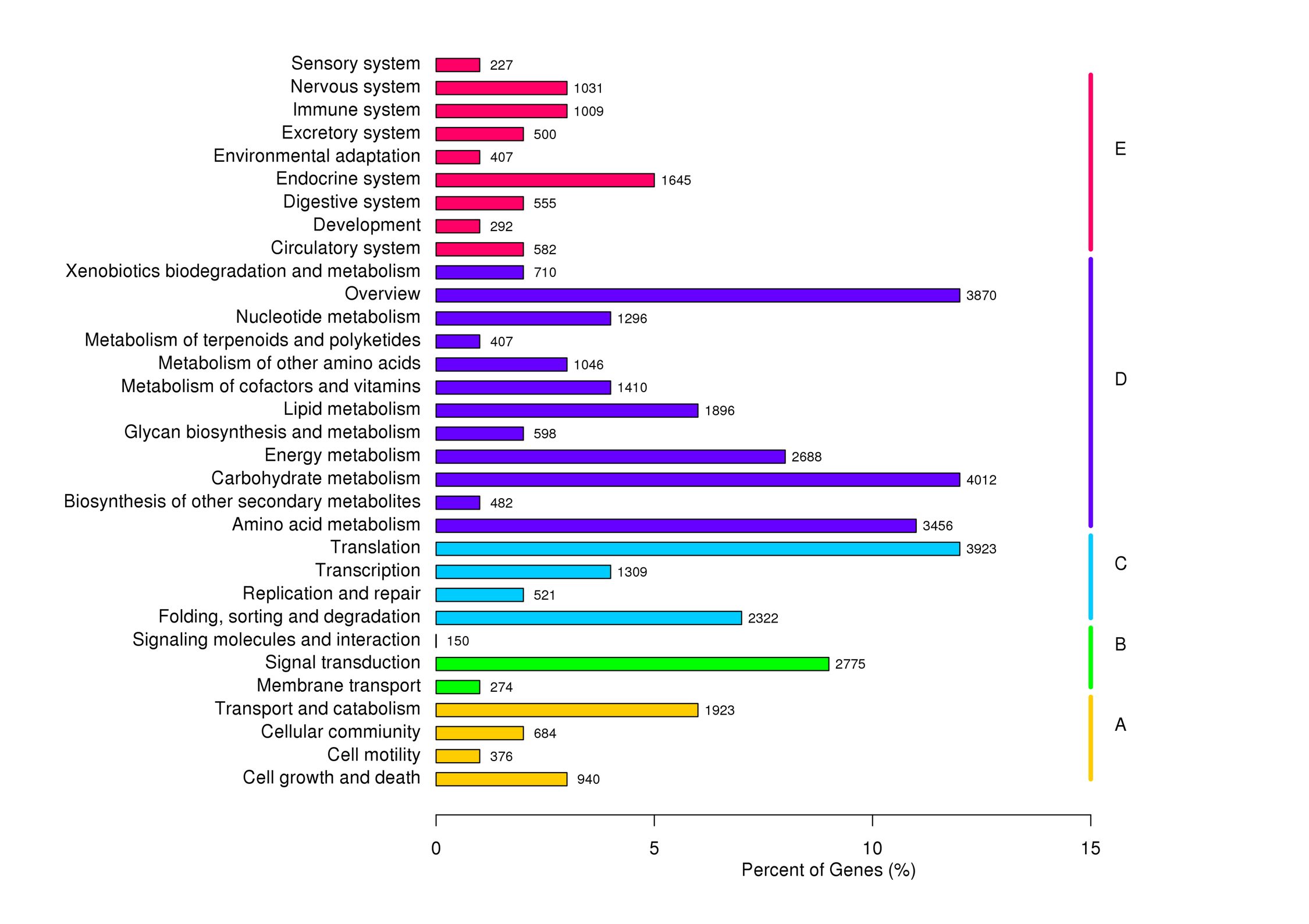

Supplement: S4 Fig — A: Cellular processes; B: Environmental information processing; C: Genetic information processing; D: Metabolism; E: Organismal systems. (TIF) [file pone.0161839.s004.tif]

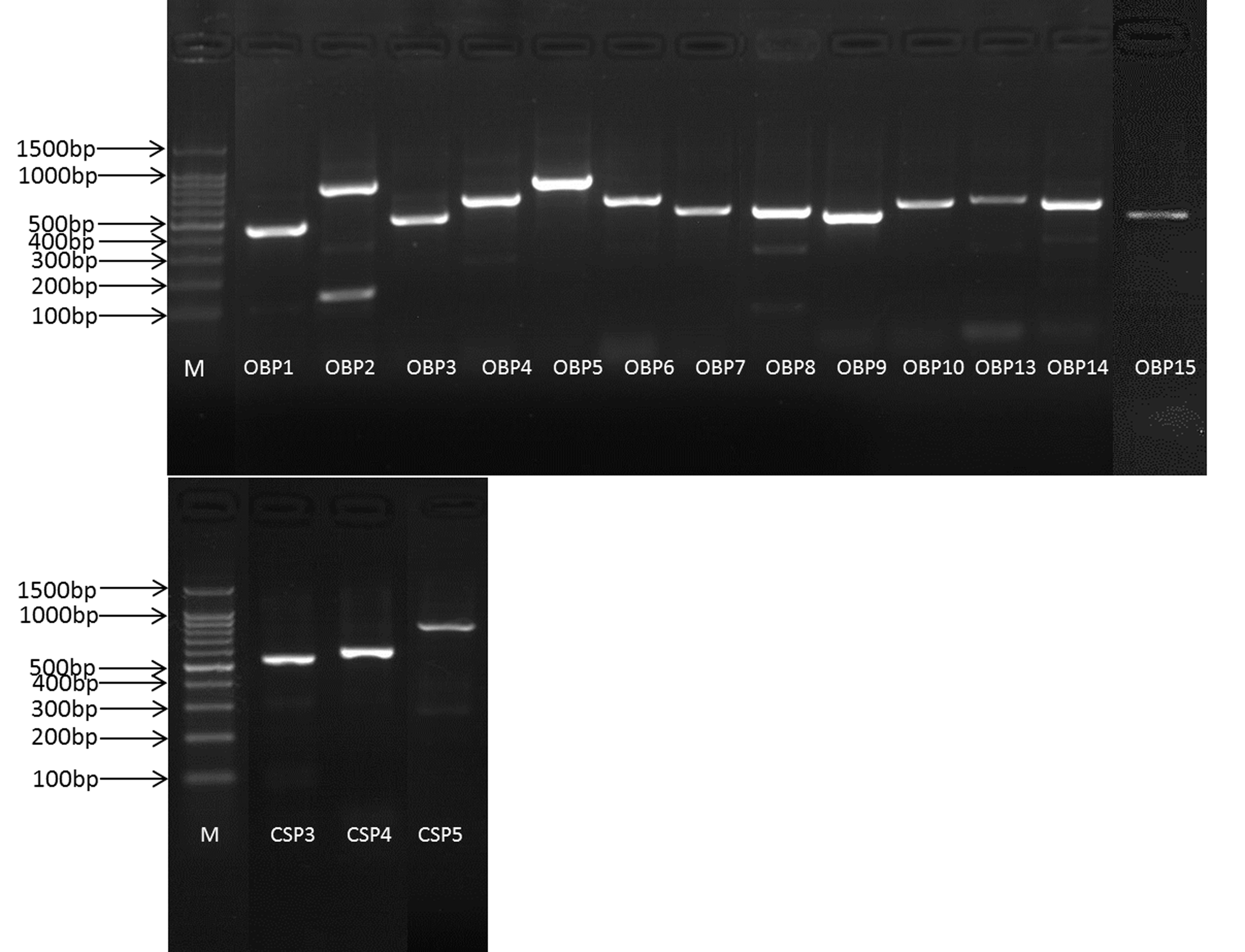

Supplement: S5 Fig — (TIF) [file pone.0161839.s005.tif]
