# Supplementary material for: Identification and Expression Analysis of Candidate Odorant-Binding Protein and Chemosensory Protein Genes by Antennal Transcriptome of Sitobion avenae
Source: PLoS One. 2016 Aug 25;11(8):e0161839. doi: 10.1371/journal.pone.0161839 (PMC4999175; doi:10.1371/journal.pone.0161839)
Supplement: S1 Table — (DOCX) [file pone.0161839.s006.docx]

**S1 Table. Primers for cloning**

| **Primer name** | **Sequences (5ˊ to 3ˊ)** |
| --- | --- |
| SaveOBP1-Forward | CCACAGGCGACCAATATCA |
| SaveOBP1-Reverse | GGAACGTAGAATGTATTACCGT |
| SaveOBP2-Forward | CTGCAGCGCGAAGACCACAGAAGTC |
| SaveOBP2-Reverse | GTATATCCATGTTTGGTTG |
| SaveOBP3-Forward | TCTGTCTGGATGCTCGTTCA |
| SaveOBP3-Reverse | CGGTCCACTCGGTTAACTCT |
| SaveOBP4-Forward | TGTGTGCGTACCAGAAGTCT |
| SaveOBP4-Reverse | GTGTTGGAATGTAAGATACAGCC |
| SaveOBP5-Forward | GTCCTCAACGAAACTGCTGC |
| SaveOBP5-Reverse | TGGTGGGATCATAACTCCGT |
| SaveOBP6-Forward | CTAGACTCTACCTAGTTGTCAC |
| SaveOBP6-Reverse | TGTCTTAACTACTACTATAGATGAGT |
| SaveOBP7-Forward | TCGTTGTCTGAAGGTCACGA |
| SaveOBP7-Reverse | AACCGGGATCTATCAGCTGC |
| SaveOBP8-Forward | AACACTATACAACTGCGGTCT |
| SaveOBP8-Reverse | GTTTACATGGCGTCGCGTC |
| SaveOBP9-Forward | ACCAGATTTGTTACAACCGAACT |
| SaveOBP9-Reverse | TGGTTGCAACTGTGTATGTGT |
| SaveOBP10-Forward | AGTTCATTGCGTTGTTGACTAC |
| SaveOBP10-Reverse | GCACAGTATGAAATTAGCGTCG |
| SaveOBP13-Forward | GACCGTGATCAGTCGTCATG |
| SaveOBP13-Reverse | GTTGAAATAACTTAATCAATCAC |
| SaveOBP14-Forward | CCCGGGGATTGTTAGTAATTTCA |
| SaveOBP14-Reverse | GCCGTACGTACACAACAACA |
| SaveOBP15-Forward | TCACCTACGTCCTACACTGT |
| SaveOBP15-Reverse | ACGACCGTCCACCAAGTATA |
| SaveCSP1-Forward | ATTTGTTGTTCTGGTCGCGT |
| SaveCSP1-Reverse | TGTTTAGCCTCTGCAGTCC |
| SaveCSP2-Forward | ATTGCACGCCATGGATTCACG |
| SaveCSP2-Reverse | GCATATAGGTACTGCATTCTGATG |
| SaveCSP3-Forward | ACTACGCTCCTCCACATCAC |
| SaveCSP3-Reverse | ATTATTGTCAGGCGTCGCAC |
| SaveCSP4-Forward | GTGACCAGTGTTATTACCACCG |
| SaveCSP4-Reverse | GCGATGGACTGGTCATGAAC |
| SaveCSP5-Forward | CAAGGACCGTGTACCAGAGA |
| SaveCSP5-Reverse | TGAGCCAAAACTTTCCCTGC |
