# Supplementary material for: Identification and Expression Analysis of Candidate Odorant-Binding Protein and Chemosensory Protein Genes by Antennal Transcriptome of Sitobion avenae
Source: PLoS One. 2016 Aug 25;11(8):e0161839. doi: 10.1371/journal.pone.0161839 (PMC4999175; doi:10.1371/journal.pone.0161839)
Supplement: S2 Table — (DOCX) [file pone.0161839.s007.docx]

**S2 Table. GenBank accession numbers of amino acid sequences used to construct the phylogenetic tree**

| **Species** | **Gene** | **Accession** |
| --- | --- | --- |
| *Pterocomma salicis* | *PsalOBP1* | CAR85660 |
|  | *PsalOBP2* | CAR85661 |
|  | *PsalOBP4* | CAR85662 |
|  | *PsalOBP9* | CAR85663 |
|  | *PsalOBP10* | CAX63261 |
| *Aphis glycines* | *AglyOBP2-AglyOBP11* | AHJ80888-AHJ80897 |
| *Aphis fabae* | *AfabOBP2* | CAR85656 |
|  | *AfabOBP8* | CAR85657 |
| *Aphis craccivora* | *AcraOBP2* | CAR85658 |
| *Brevicoryne brassicae* | *BrebrOBP3* | AEX65667 |
| *Tuberolachnus salignus* | *TsalOBP1* | CAR85659 |
| *Drepanosiphum platanoidis* | *DplaOBP3* | AEX65663 |
| *Lipaphis erysimi* | *LeryOBP3* | AJO61166 |
|  | *LeryOBP7* | AJO61167 |
| *Megoura viciae* | *MvicOBP1* | CAR85650 |
|  | *MvicOBP2* | CAR85651 |
|  | *MvicOBP5* | CAR85652 |
|  | *MvicOBP8* | CAR85653 |
|  | *MvicOBP10* | CAX63260 |
| *Metopolophium dirhodum* | *MdirOBP1* | CAR85638 |
|  | *MdirOBP2* | CAR85639 |
|  | *MdirOBP3* | CAX63256 |
|  | *MdirOBP4* | CAR85640 |
|  | *MdirOBP5* | CAR85641 |
|  | *MdirOBP6* | CAR85642 |
|  | *MdirOBP8* | CAR85643 |
| *Myzus persicae* | *MperOBP3* | CAR85644 |
|  | *MperOBP4* | CAR85645 |
|  | *MperOBP6* | CAR85646 |
|  | *MperOBP7* | CAR85647 |
|  | *MperOBP8* | CAR85648 |
|  | *MperOBP10* | CAR85649 |
|  | *MperCSP1* | ACJ64047 |
|  | *MperCSP4* | ACJ64048 |
|  | *MperCSP5* | ACJ64049 |
| *Nasonovia ribis-nigri* | *NribOBP2* | CAR85654 |
|  | *NribOBP3* | CAX63257 |
|  | *NribOBP5* | CAX63258 |
|  | *NribOBP7* | CAX63259 |
|  | *NribOBP8* | CAR85655 |
| *Rhopalosiphum padi* | *RpadOBP2* | CAX63253 |
|  | *RpadOBP5* | CAX63254 |
|  | *RpadOBP10* | CAX63255 |
| *Aphis gossypii* | *AgosOBP2-10* | AGE97632-AGE97640 |
|  | *AgosCSP1* | AGE97641 |
|  | *AgosCSP2* | AGE97642 |
|  | *AgosCSP4* | AGE97643 |
|  | *AgosCSP5* | AGE97644 |
|  | *AgosCSP6* | AGE97645 |
|  | *AgosCSP9* | AGE97648 |
|  | *AgosCSP10* | AGE97649 |
| *Acyrthosiphon pisum* | *ApisOBP1-AipsOBP10* | CAR85628-CAR85637 |
|  | *ApisOBP11-13* | CAX63068-CAX63070 |
| *Apolygus lucorum* | *AlucOBP1-2* | AEA07705-AEA07706 |
|  | *AlucOBP3-6* | AEA07661-AEA07664 |
|  | *AlucOBP7-12* | AFJ54048- AFJ54053 |
|  | *AlucCSP1-8* | AGD80081-AGD80088 |
| *Adelphocoris lineolatus* | *AlinOBP1-6* | ACZ58027-ACZ58032 |
|  | *AlinOBP7* | ACZ58085 |
|  | *AlinOBP8-13* | ACZ58079-ACZ58085 |
|  | *AlinCSP1-AlinCSP8* | ACZ58019-ACZ58026 |
| *Sogatella furcifera* | *SfurOBP1-10* | AGZ04901- AGZ04910 |
|  | *SfurOBP11* | AHB59662 |
|  | *SfurOBP12* | AHJ61048 |
|  | *NlugCSP1- NlugCSP9* | AGZ04911- AGZ04919 |
| *Nilaparvata lugens* | *NlugOBP1- NlugOBP3* | ACI30679- ACI30681 |
|  | *NlugOBP4- NlugOBP8* | AGZ04895-AGZ04899 |
|  | *NlugOBP9* | AGO81742 |
|  | *NlugOBP10* | AGZ04900 |
|  | *NlugCSP1- NlugCSP2* | ADN06871-ADN06872 |
|  | *NlugCSP3- NlugCSP6* | ACJ64050- ACJ64053 |
|  | *NlugCSP7* | ADN06873 |
|  | *NlugCSP8- NlugCSP9* | ACJ64054- ACJ64055 |
|  | *NlugCSP10* | AGZ04900 |
|  | *NlugCSP11* | AGO81736 |
