# Supplementary material for: Identification and Expression Analysis of Candidate Odorant-Binding Protein and Chemosensory Protein Genes by Antennal Transcriptome of Sitobion avenae
Source: PLoS One. 2016 Aug 25;11(8):e0161839. doi: 10.1371/journal.pone.0161839 (PMC4999175; doi:10.1371/journal.pone.0161839)
Supplement: S3 Table — (DOCX) [file pone.0161839.s008.docx]

**S3 Table. Primers for qPCR**

| **Primer name** | **Sequences (5ˊto 3ˊ)** |
| --- | --- |
| SaveOBP1P-Forward | TGAATTTCTACGGGGCGGTA |
| SaveOBP1P-Reverse | CACACTCTTCAGTCGGCAAG |
| SaveOBP2P-Forward | CATCAAGTCCGGTGCCAATG |
| SaveOBP2P-Reverse | GAGCAACATGTCCTTCGGTC |
| SaveOBP3P-Forward | ACCATCGACAGAAACCGGAA |
| SaveOBP3P-Reverse | CGTAGACCATTCCGACCAGT |
| SaveOBP4P-Forward | ACGCCAAGTCATAGCAGTAGA |
| SaveOBP4P-Reverse | ATCAAGCCGTCTACTACCGG |
| SaveOBP5P-Forward | CAGTGGCAGAAACAAGTGGG |
| SaveOBP5P-Reverse | CCTGTACCGGTTTCTCGTCT |
| SaveOBP6P-Forward | TTGCGATCATCTGCCAAACA |
| SaveOBP6P-Reverse | CCGGGCTTGGAATGAGAGTT |
| SaveOBP7P-Forward | GAGTGAAGCGGCCATTAAAA |
| SaveOBP7P-Reverse | TGCCATCATCGTCATCTTGT |
| SaveOBP8P-Forward | CGACCAAGGTTGCACAGAAA |
| SaveOBP8P-Reverse | AGTAGTCACAATTCCGGCCA |
| SaveOBP9P-Forward | ACCTGCGAAGTTCCTCGAAT |
| SaveOBP9P-Reverse | GTTCTTTCAGTGCTGGCGAT |
| SaveOBP10P-Forward | ACACGACCACAACCAGATGA |
| SaveOBP10P-Reverse | ACATCTCGTCGAAGCAACAC |
| SaveOBP13P-Forward | GACACTCTCGAGCTGGACT |
| SaveOBP13P-Reverse | AGCCGGCAGAGTTGATTTTG |
| SaveOBP14P-Forward | CGTTTGCGGCGAATTGTTAT |
| SaveOBP14P-Reverse | TTCCACTTGCCGGTATCCTT |
| SaveOBP15P-Forward | CATCGCGTCACTCCAGAAAG |
| SaveOBP15P-Reverse | GTCTCGCCATCAACTGTTCC |
| SaveCSP1P-Forward | ATTTGTTGTTCTGGTCGCGT |
| SaveCSP1P-Reverse | TGGCCTTCCTCGGTACAATT |
| SaveCSP2P-Forward | GTAGTCCCACCACCTACACC |
| SaveCSP2P-Reverse | TCTAAGTTCAGCTCCTTCGGG |
| SaveCSP3P-Forward | CGAAAGGCAAATGACCACCA |
| SaveCSP3P-Reverse | ACGCGTCGAGGAATTTGTTC |
| SaveCSP4P-Forward | CCACTTCCGACGACCATAGT |
| SaveCSP4P-Reverse | CTTTGCACGAACCTCCTGTC |
| SaveCSP5P-Forward | AACCACCAGAGAAACGTCGT |
| SaveCSP5P-Reverse | TGAGCTCCAAACCCTCTCTG |
| NADH-Forward | CGAGGAGAACATGCTCTTAGAC |
| NADH-Reverse | GATAGCTTGGGCTGGACATATAG |
| Actin-Forward | CGTTACCAACTGGGACGATATG |
| Actin-Reverse | GGGTTCAATGGAGCTTCTGTTA |
