# Supplementary material for: Identification and Expression Analysis of Candidate Odorant-Binding Protein and Chemosensory Protein Genes by Antennal Transcriptome of Sitobion avenae
Source: PLoS One. 2016 Aug 25;11(8):e0161839. doi: 10.1371/journal.pone.0161839 (PMC4999175; doi:10.1371/journal.pone.0161839)
Supplement: S4 Table — (DOCX) [file pone.0161839.s009.docx]

**S4 Table . Success rate of gene annotation**

| **Annotation source** | **Number of unigenes** | **Percentage (%)** |
| --- | --- | --- |
| NR | 89425 | 67.06 |
| NT | 42393 | 31.79 |
| KO | 32300 | 24.22 |
| SwissProt | 55639 | 41.72 |
| PFAM | 54572 | 40.92 |
| GO | 60371 | 45.27 |
| KOG | 39254 | 29.44 |
| All databases | 13788 | 10.34 |
| At least one database | 100345 | 75.26 |
| Total Unigenes | 133331 | 100 |
