# Supplementary material for: Identification and Expression Analysis of Candidate Odorant-Binding Protein and Chemosensory Protein Genes by Antennal Transcriptome of Sitobion avenae
Source: PLoS One. 2016 Aug 25;11(8):e0161839. doi: 10.1371/journal.pone.0161839 (PMC4999175; doi:10.1371/journal.pone.0161839)
Supplement: S5 Table — Calculations are based on amino acid sequence alignment by DNAMAN. The percentage identity for each pair is shown. (DOCX) [file pone.0161839.s010.docx]

**S5 Table. Sequence identity between SaveOBPs**

|  | **SaveOBP1** | **SaveOBP2** | **SaveOBP3** | **SaveOBP4** | **SaveOBP5** | **SaveOBP6** | **SaveOBP7** | **SaveOBP8** | **SaveOBP9** | **SaveOBP10** | **SaveOBP13** | **SaveOBP14** | **SaveOBP15** |
| --- | --- | --- | --- | --- | --- | --- | --- | --- | --- | --- | --- | --- | --- |
| **SaveOBP1** | 100 |  |  |  |  |  |  |  |  |  |  |  |  |
| **SaveOBP2** | 8.5 | 100 |  |  |  |  |  |  |  |  |  |  |  |
| **SaveOBP3** | 12.24 | 7.82 | 100 |  |  |  |  |  |  |  |  |  |  |
| **SaveOBP4** | 16.00 | 10.29 | 7.5 | 100 |  |  |  |  |  |  |  |  |  |
| **SaveOBP5** | 12.67 | 11.79 | 17.19 | 14.48 | 100 |  |  |  |  |  |  |  |  |
| **SaveOBP6** | 11.57 | 12.30 | 13.20 | 17.35 | 14.72 | 100 |  |  |  |  |  |  |  |
| **SaveOBP7** | 16.56 | 9.43 | 13.64 | 9.05 | 11.76 | 12.56 | 100 |  |  |  |  |  |  |
| **SaveOBP8** | 30.64 | 12.69 | 17.68 | 12.02 | 11.31 | 10.70 | 13.10 | 100 |  |  |  |  |  |
| **SaveOBP9** | 11.45 | 11.11 | 12.65 | 13.43 | 14.48 | 16.28 | 17.86 | 17.41 | 100 |  |  |  |  |
| **SaveOBP10** | 14.29 | 11.93 | 13.33 | 13.07 | 10.41 | 10.65 | 27.52 | 10.18 | 12.56 | 100 |  |  |  |
| **SaveOBP13** | 11.84 | 6.58 | 7.09 | 11.56 | 9.05 | 8.37 | 10.74 | 11.73 | 8.43 | 11.89 | 100 |  |  |
| **SaveOBP14** | 12.43 | 9.88 | 14.37 | 13.57 | 13.90 | 17.51 | 16.57 | 17.34 | 18.60 | 12.50 | 13.17 | 100 |  |
| **SaveOBP15** | 12.73 | 9.84 | 14.20 | 9.55 | 12.22 | 13.02 | 11.80 | 15.73 | 13.63 | 15.91 | 6.21 | 20.00 | 100 |

Calculations are based on the amino acid sequence alignment done by DNAMAN. The percentage of identity of each pair is shown.
