# Supplementary material for: Identification and Expression Analysis of Candidate Odorant-Binding Protein and Chemosensory Protein Genes by Antennal Transcriptome of Sitobion avenae
Source: PLoS One. 2016 Aug 25;11(8):e0161839. doi: 10.1371/journal.pone.0161839 (PMC4999175; doi:10.1371/journal.pone.0161839)
Supplement: S6 Table — Calculations are based on amino acid sequence alignment by DNAMAN. The percentage identity of each pair is shown. (DOCX) [file pone.0161839.s011.docx]

**S6 Table. Sequence identity between SaveCSPs**

|  | **SaveCSP1** | **SaveCSP2** | **SaveCSP3** | **SaveCSP4** | **SaveCSP5** |
| --- | --- | --- | --- | --- | --- |
| **SaveCSP1** | 100 |  |  |  |  |
| **SaveCSP2** | 30.82 | 100 |  |  |  |
| **SaveCSP3** | 28.57 | 20.13 | 100 |  |  |
| **SaveCSP4** | 11.89 | 19.21 | 17.85 | 100 |  |
| **SaveCSP5** | 15.22 | 18.26 | 17.39 | 12.61 | 100 |

Calculations are based on the amino acid sequence alignment done by DNAMAN. The percentage of identity of each pair is shown.
